# Solid-state fermentation in multi-well plates to assess pretreatment efficiency of rot fungi on lignocellulose biomass

**DOI:** 10.1111/1751-7915.12307

**Published:** 2015-08-06

**Authors:** Simeng Zhou, Sana Raouche, Sacha Grisel, David Navarro, Jean-Claude Sigoillot, Isabelle Herpoël-Gimbert

**Affiliations:** 1 INRA, UMR 1163 Biodiversity and Biotechnology of FungiF-13009, Marseille, France; 2 Aix-Marseille Université, Polytech Marseille, UMR 1163 Biodiversity and Biotechnology of FungiF-13009, Marseille, France; 3 International Centre for Microbial Resources collection-Filamentous Fungi, CIRM-CFF-13009, Marseille, France

## Abstract

The potential of fungal pretreatment to improve fermentable sugar yields from wheat straw or Miscanthus was investigated. We assessed 63 fungal strains including 53 white-rot and 10 brown-rot fungi belonging to the Basidiomycota phylum in an original 12 day small-scale solid-state fermentation (SSF) experiment using 24-well plates. This method offers the convenience of one-pot processing of samples from SSF to enzymatic hydrolysis. The comparison of the lignocellulolytic activity profiles of white-rot fungi and brown-rot fungi showed different behaviours. The hierarchical clustering according to glucose and reducing sugars released from each biomass after 72 h enzymatic hydrolysis splits the set of fungal strains into three groups: efficient, no-effect and detrimental-effect species. The efficient group contained 17 species belonging to seven white-rot genera and one brown-rot genus. The yield of sugar released increased significantly (max. 62%) compared with non-inoculated controls for both substrates.

## Introduction

Fungi play a dominant role in lignocellulose conversion. Wood-decay fungi, including white-rot and brown-rot fungi (WRFs and BRFs), have been extensively studied for their abilities to efficiently modify, degrade and depolymerize major plant cell wall components. Among them, some WRFs are able to mineralize the more recalcitrant lignin with little consumption of cellulose (Martinez *et al*., 2005). This unique ability lends great potential interest for them in the pretreatment of biomass in many biotechnological applications, such as wood pulping and bleaching, and biofuel production.

Solid-state fermentation (SSF) is defined by Barrios-Gonzalez (2012) as when a microbial culture develops on the surface and inside of a solid matrix in the absence of free water. SSF has been shown to be of interest in at least two types of applications: the production of lignocellulolytic enzymes and the degradation of lignocellulose itself. SSF mimics the natural habitat of the fungus to facilitate enzyme secretion. Industrial enzymes for biofuel applications have been successfully produced at the commercial level by SSF (Singhania *et al*., 2010), and differences in enzyme production by fungus between SSF and submerged fermentation (SmF) have been reviewed by Barrios-Gonzalez (2012). Enzymes are generally produced in much higher concentrations in SSF, some of them display higher optimal temperature or pH stability and some are secreted only in SSF. Regarding the degradation of lignocellulose, the enzymes secreted along mycelia are close to their substrates, and the rapid diffusion of oxygen in SSF creates favourable conditions to achieve high performance in the oxidative degradation of lignocellulose. These findings have led to fungi to be considered as a suitable alternative for lignocellulose pretreatment in the second-generation ethanol production processes due to their low cost, environmental friendliness and adaptability to both continuous and batch processes. Moreover, cellulose and hemicellulose become more accessible for hydrolysis by the breakdown of the lignin–carbohydrate complexes, and so avoid substantial holocellulose (cellulose and hemicellulose) loss, thereby increasing conversion rates and productivity of fermentable sugar. Recent studies have demonstrated the efficiency of some fungal strains [e.g. *Ceriporiopsis subvermispora* (Cianchetta *et al*., 2014), *Pycnoporus cinnabarinus* (Gupta *et al*., 2011), *Trametes hirsuta* (Saritha *et al*., 2012), *Irpex lacteus* (Salvachua *et al*., 2013), *Gloeophyllum trabeum* and *Postia placenta* (Schilling *et al*., 2012)] in such pretreatment steps for improving the production of bioethanol or biogas from various substrates. Two distinguishable patterns of biomass breakdown, namely simultaneous lignin and polysaccharide degradation or selective delignification have been described among WRFs (Martinez *et al*., 2005). The simultaneous attack of cellulose and lignin used as a preferred strategy by some strains could result in the concurrent production of cellulases, hemicellulases and ligninolytic activities; whereas fungal strains acting as selective delignifiers secrete very low levels of holocellulolytic enzymes. However, selective delignification ranges among fungal species according to pretreatment time and the type of lignocellulosic biomass (Wan and Li, 2010).

A biodiversity screening step is a prerequisite in choosing the best fungus for efficient pretreatment. Natural fungal diversity offers a huge array of new fungal strains with high biotechnological potential (Blackwell, 2011; Berrin *et al*., 2012). Fungal biodiversity has been investigated previously for biomass pretreatment, but these studies were often restricted to a limited number of strains (Li *et al*., 2008; Salvachua *et al*., 2011). Traditional screening is performed with multiple fungal SSF in flasks and it is difficult to apply these culture conditions to large-scale strain screening. Scaled-down screening methods hold great promise for accelerating progress in biomass conversion as they lead to the selection of a narrower number of most effective strains to be later tested in a scaled-up process. This strategy has been successfully used in screening the transformation of various biomasses (Santoro *et al*., 2010) and biomass-degrading enzymes (Navarro *et al*., 2010; Cianchetta *et al*., 2012).

In this paper, we screened biodiversity for fungal pretreatment of lignocellulosic biomasses to preselect promising candidates, based on the yields of fermentable sugar, for scaled-up fermentation. For this purpose, 63 strains of basidiomycetes were grown on wheat straw or on miscanthus using a medium-throughput SSF screening method. The ligninolytic and carbohydrate hydrolytic activities involved in biomass degradation were also examined.

## Results and discussion

### 24-well plate-based screening of basidiomycete strains

Biological pretreatment using wood-rotting fungi is a well-researched process for bioethanol production from lignocellulosic materials. The main fungal groups of interest for lignocellulose degradation are the WRFs and the BRFs belonging to the Basidiomycota phylum (Martinez *et al*., 2005).

**Figure 1:**
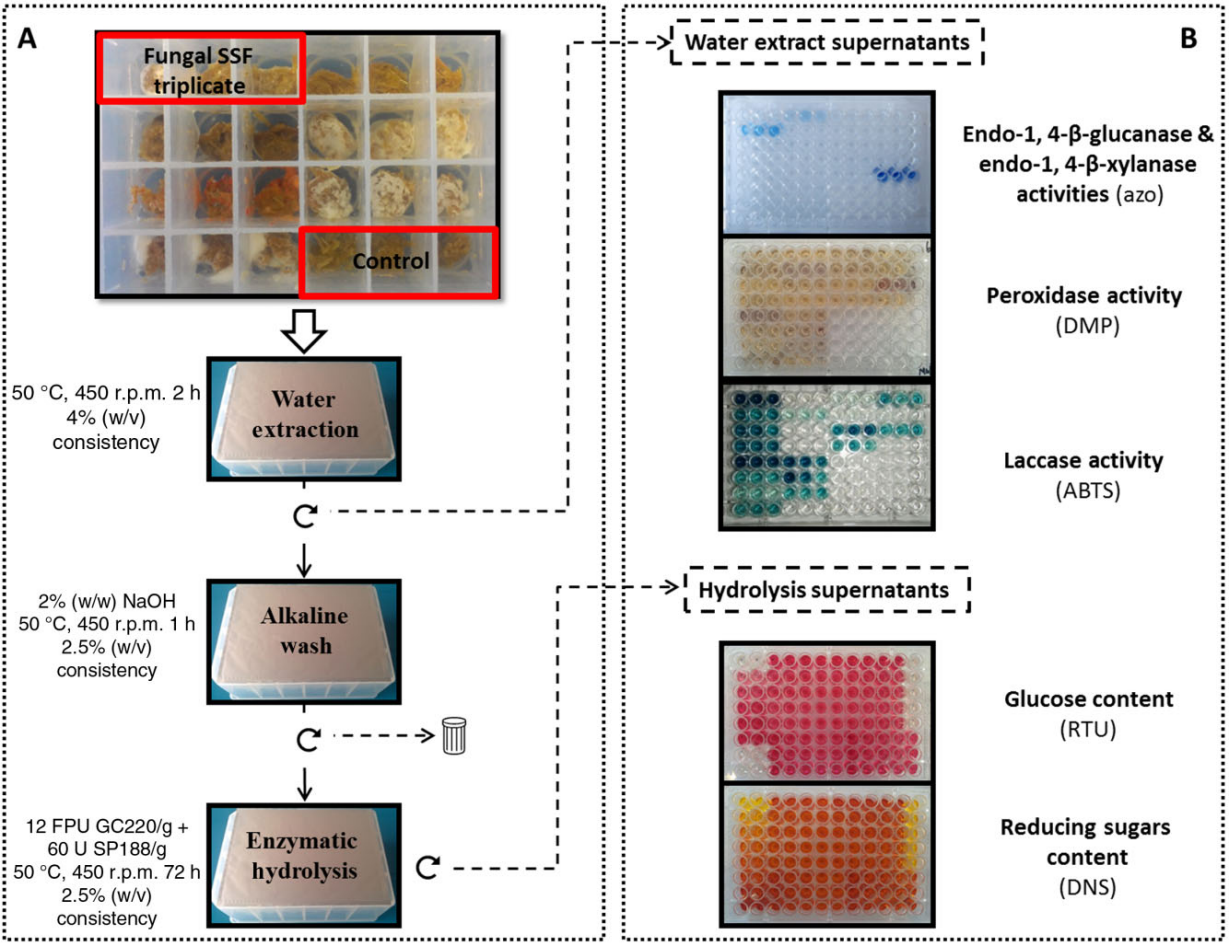
Experimental procedure: (A) fungal SSF pretreatment and sample processing; (B) analysis. Wells were inoculated in triplicate for each fungal strain. After 12 days SSF in a 24-well plate, substrates underwent *in situ* sequential sample processing to assess water-soluble components and sugars released after enzymatic hydrolysis with a commercial cellulase cocktail of *T. reesei* (GC 220, Genencor) and β-glucosidase from *A. niger* (SP 188, Novozymes). (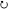) centrifugation step, (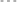) supernatant fraction, (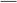) solid fraction, (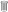) discarded fraction.

The new 24-well plate-based screening method was used to evaluate the potential of several strains of basidiomycetes simultaneously (Fig. 1). SSF of lignocellulosic substrates and subsequent enzymatic hydrolysis of the pretreated biomasses were performed. This allows one-pot multistep treatments including fungal growth, mild alkaline wash and enzymatic hydrolysis. This procedure was refined from to an experimental design previously described in studies on the evaluation of biological pretreatment (Tian *et al*., 2012; Lopez-Abelairas *et al*., 2013). A culture time of 12 days combined with a high inoculum loading allowed extensive and fast fungal colonization. The mild alkaline wash performed before enzymatic hydrolysis is reported to improve the enzymatic digestibility of the biopretreated substrate (Yu *et al*., 2010; Salvachua *et al*., 2011). The main effect of sodium hydroxide pretreatment on lignocellulosic biomass was shown to be delignification through the cleavage of the ester bonds cross-linking lignin and xylan, thus increasing the porosity of the biomass (McIntosh and Vancov, 2011; Maurya *et al*., 2015). In addition, fungal hyphae grow on the surface of the lignocellulosic materials forming a dense hyphal coat that hinders cellulases' access to cellulose. As suggested for washing or heating steps before enzymatic hydrolysis, the very mild alkaline conditions applied were expected to remove this biological barrier or inhibitors of saccharification (Shi *et al*., 2009). The additional effects of these actions were expected to render the biomass more susceptible and accessible to saccharification, and increase the production of fermentable sugar.

Compared with traditional cultures grown in flasks or in packed columns (bioreactors), the 24-well plate-based method requires less ‘hands-on’ time and allows the simultaneous processing of multiple strains. It is therefore well adapted to screen a large number of fungi. Additionally, this method avoids excess substrate handling and losses, thus favouring a more accurate analysis.

In this study, a total of 63 fungal strains comprising 53 WRFs and 10 BRFs were screened on wheat straw and miscanthus. The most effective strains for biopretreatment should consume little sugar for their own growth while promoting a high lignocellulose deconstruction to enhance enzymatic hydrolysis. Among those strains examined, 58 belonged to *Polyporales*, mainly from *Polyporaceae* (32), *Ganodermataceae* (13) and *Fomitopsidaceae* (10), representing the distribution of fungal diversity in the International Centre of Microbial Resources (CIRM-CF) collection (Table S1). Unlike the WRFs, the BRFs were exclusively collected from temperate forests: these fungi, described as less deeply involved in the decay of hardwood compared with softwood, dominate the decomposition of conifer wood in boreal forests (Eastwood *et al*., 2011). Some strains of the same species were selected to compare their performance in pretreatment according to the geographical zone where they were collected.

### Enzymatic hydrolysis of fungal pretreated lignocellulosic biomass

Visual observation of the cultures indicated mycelial growth of all the strains regardless of the substrate. To evaluate the effect of fungal pretreatment on saccharification, released reducing sugars and glucose from pretreated miscanthus and wheat straw were determined after 72 h of enzymatic hydrolysis using cellulases of *Trichoderma reesei* supplemented with a β-glucosidase from *Aspergillus niger*.

**Figure 2:**
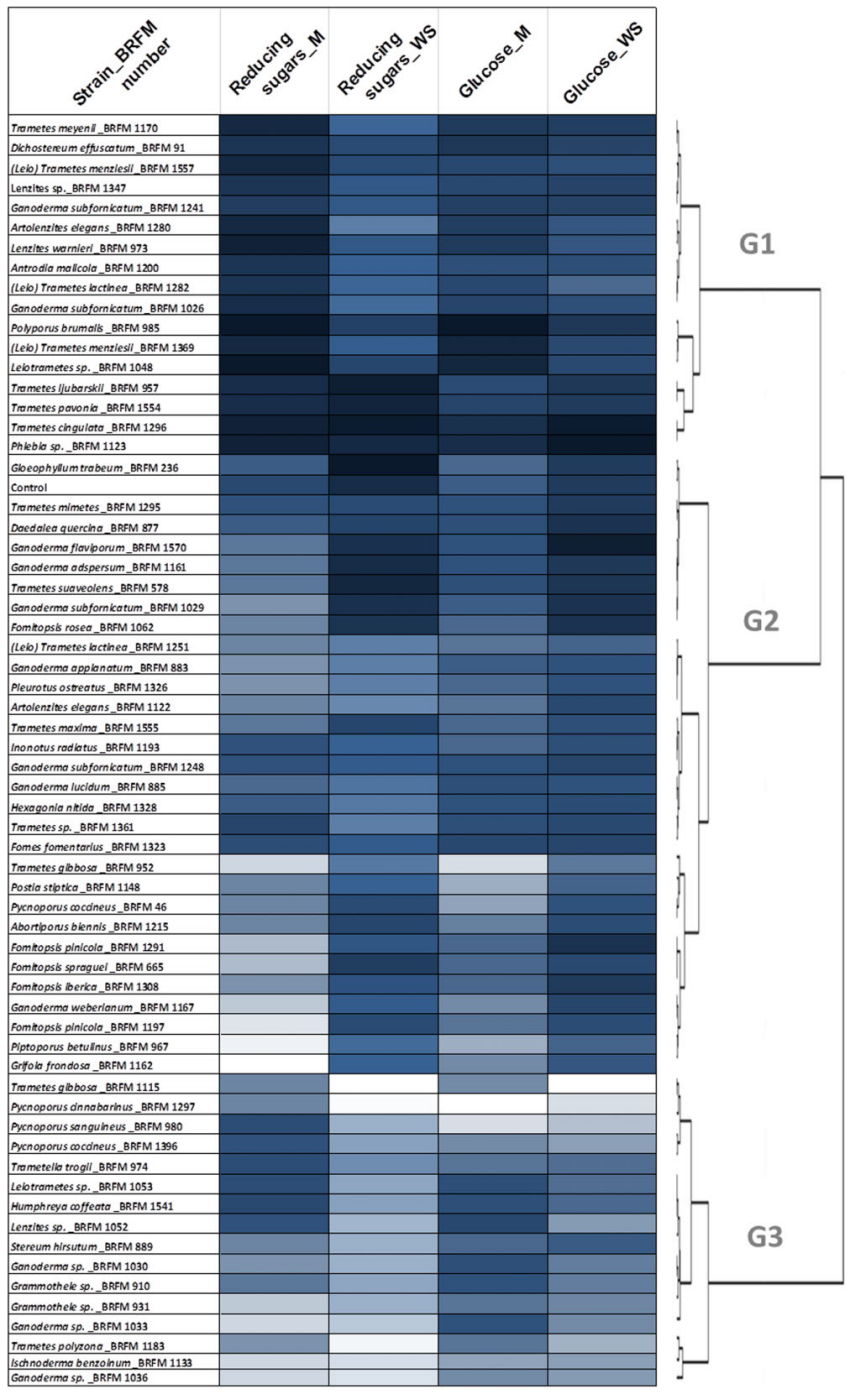
Hierarchical cluster analysis (HCA) of sugar release after enzymatic hydrolysis of fungal pretreated wheat straw (WS) and miscanthus (M). HCA was performed on PCA coordinates in two-dimensional space ensuring consideration of 91% of the total variation. Clusters were defined as: efficient (G1), no-effect (G2) and detrimental-effect species (G3) compared with the control. Each value is the mean of the three replicates.

A hierarchical cluster analysis (HCA) was performed to characterize the grouping of fungal species according to their efficiency in enhancing enzymatic hydrolysis. A principal component analysis (PCA) was carried out prior to HCA to better highlight the main feature of the data set. The first two principal components showed the highest eigenvalues and accounted for 91% of the total variation; they were kept for the clustering. HCA was performed by calculating the mean Euclidian distances between the samples, followed by a hierarchical tree construction using Ward's criterion. The fungal species were split into three major groups according to their ability to improve enzymatic hydrolysis (Fig. 2): efficient (G1), no-effect (G2) and detrimental-effect (G3) species compared with the control.

We noted that the 11 strains of *Trametes*, the 12 strains of *Ganoderma* and the six strains of *Leiotrametes* fell into different groups, while five strains of the *Fomitopsis* genus did not. Intraspecific differences were also globally observed for several strains of the same species, regardless of their geographic origin. These results are consistent with previous works (Elisashvili *et al*., 2008; Simonic *et al*., 2010; Berrin *et al*., 2012), indicating intrageneric and intraspecific differences of efficiency in enhancing lignocellulosic biomass bioconversion.

The fungal group giving the highest saccharification results (G1) included the genera *Trametes* (eight strains), *Polyporus*, *Phlebia*, *Ganoderma*, *Lenzites* (two strains), *Dichostereum*, *Antrodia* and *Artolenzites*. A noteworthy feature of the G1 strains was their remarkable ability to improve saccharification of miscanthus. Fungal pretreatment of this recalcitrant crop appears to be especially useful and could, in combination with the existing conventional physicochemical treatment, permit less drastic operating conditions than those currently required (Keller *et al*., 2003; Wang *et al*., 2010; 2012; Li and Chen, 2014). Of these strains, *Polyporus brumalis* BRFM 985, *Trametes menziesii* BRFM 1369, *Leiotrametes sp.* BRFM 1048, *Trametes ljubarskii* BRFM 957, *Trametes pavonia* BRFM 1554, *Trametes cingulata* BRFM 1296 and *Phlebia sp.* BRFM 1123 proved to be particularly efficient in increasing the yields of fermentable sugar from wheat straw and miscanthus with notably a maximum improvement of up to 15% and 62% of glucose, respectively, compared with the control. Most of the genera in the cluster G1 (*Polyporus*, *Trametes*, *Ganoderma*, *Antrodia*, *Lenzites* and *Phlebia*) have already been reported to show potential for application in the biological pretreatment of several feedstocks (Saritha *et al*., 2012; Vaidya and Singh, 2012; Wang *et al*., 2012; Ryu *et al*., 2013; Deswal *et al*., 2014). We note that the sole remaining fungal genus identified in this work as being able to increase enzymatic hydrolysis of miscanthus, i.e. *Dichostereum*, has to our knowledge not yet been explored. Also, strains belonging to the genera *Pycnoporus*, *Pleurotus* and *Gloeophyllum* had previously been identified as good candidates for the bioprocessing of switchgrass, wheat straw and aspen, respectively (Schilling *et al*., 2012; Liu *et al*., 2013; Lopez-Abelairas *et al*., 2013), but were not highlighted in our screening. The discrepancy between our results and those of the literature could arise from: (i) strain differences at both inter- and intraspecific levels, (ii) culture conditions and (iii) nature of the substrate.

The concept behind biomass degradation by fungi is the secretion of a complex mixture of cell wall-degrading enzymes, including cellulases, hemicellulases and lignin-degrading enzymes. Fungi with high selectivity for lignin degradation, thereby leading to less holocellulose loss during growth, are important for a successful fungal pretreatment. The data provided by genomic, transcriptomic and secretomic analyses of wood-decay fungi show that the fungal enzymatic system and non-enzymatic oxidative mechanisms involved in lignocellulose depolymerization form a complex process that depends on many factors such as substrates, fungal strain and culture conditions (Vanden Wymelenberg *et al*., 2010; Grigoriev *et al*., 2011). Owing to differences in the chemical composition and structure of the substrates to be treated, the strains suitable for one substrate may not be always suitable for others. It is therefore essential to select the best-adapted fungal strain for a given substrate. This underlines the crucial importance of a biodiversity exploration step to define the best fungal strain/biomass combination to develop an efficient bioprocess. This study also reminds us that fungal biodiversity is a huge underexploited asset for innovation. It offers opportunities for the use of unexplored fungi with biotechnological potential in biorefining, such as the *Dichostereum* genera, which was highlighted as being efficient in pretreating miscanthus.

### Lignocellulolytic enzyme profiles during fungal SSF

To gain insight into the lignocellulose-degrading enzymes secreted during fungal pretreatment, pattern and levels of ligninolytic and carbohydrate hydrolytic activities were analysed in the water-soluble extracts from the 12-day-old cultures of fungal strains growing on both substrates. Laccase and peroxidases related to lignin degradation, and the activities of endo-1,4-β-glucanase and endo-1,4-β-xylanase, key enzymes in the degradation of holocellulose, were determined.

**Figure 3:**
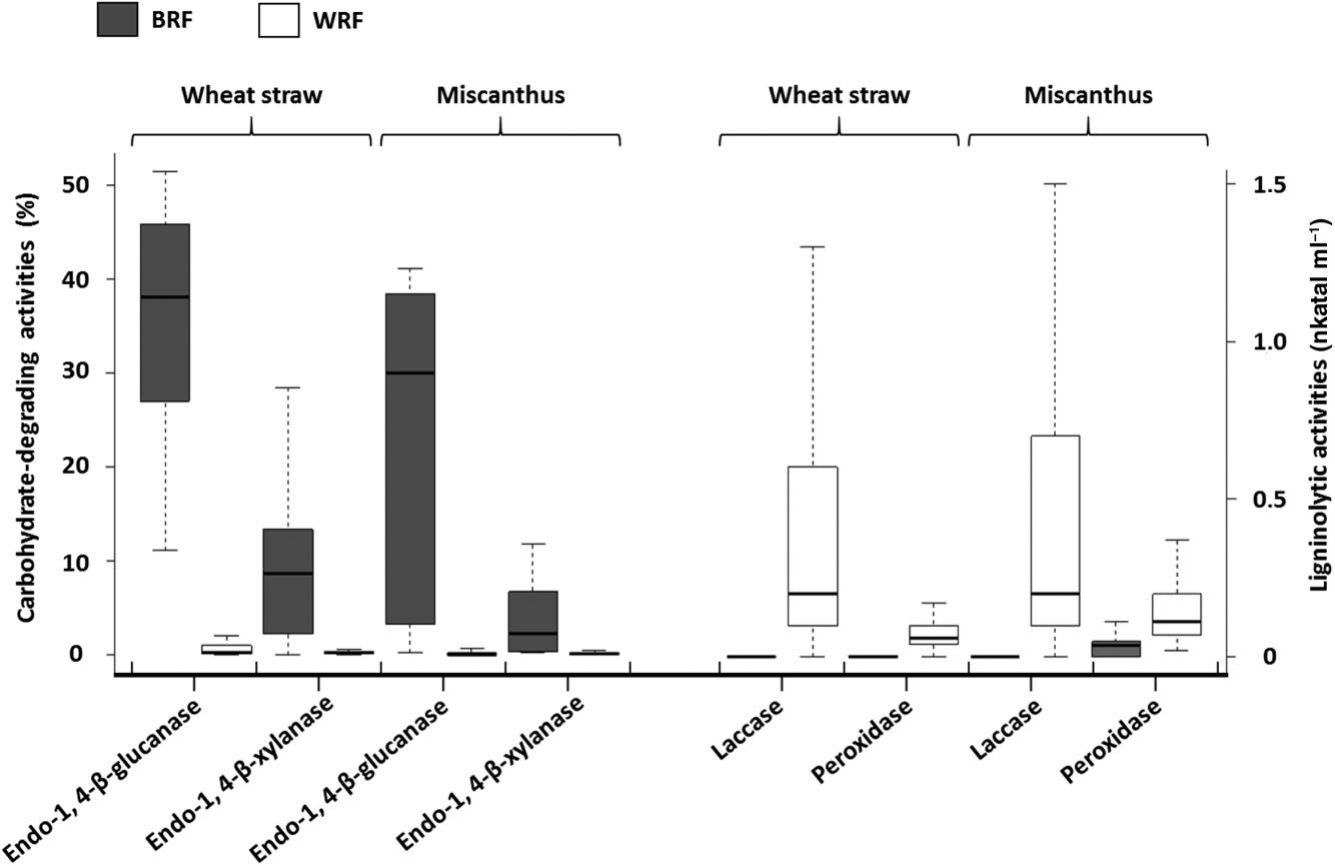
Pattern and levels of ligninolytic and carbohydrate-hydrolysing activities of BRFs and WRFs after 12 days of solid-state fermentation on wheat straw or miscanthus.

In general, a low or zero level of endo-1,4-β-glucanase and endo-1,4-β-xylanase was found in the WRF cultures, whereas most of the BRFs tested produced high levels of carbohydrate hydrolytic activities on both substrates (Fig. 3). The highest production of hydrolytic activities found in BRF cultures, as described elsewhere (Machuca and Ferraz, 2001; Mathieu *et al*., 2013), could be explained by a rapid and extensive holocellulose depolymerization in the early stages of the decay process (Arantes *et al*., 2012). In addition, laccase and peroxidase activities were found mainly in the water extracts of WRF cultures (Fig. 3). In contrast to WRFs, most BRFs were shown to lack extracellular phenoloxidases, although a few of them have been reported to produce peroxidases (Huang *et al*., 2009; Yadav *et al*., 2011). It is known that BRFs modify lignin slightly, mostly by demethoxylation occurring through mediated Fenton reactions (Arantes *et al*., 2012).

**Figure 4:**
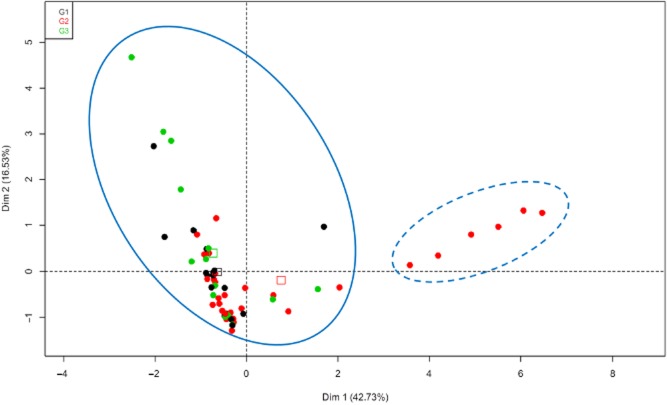
Ligno- and holocellulolytic activities cluster plot. HCA was performed on PCA coordinates in four-dimensional space ensuring consideration of 83% of the total variation. Data were clustered in two groups represented by two ellipses (straight line and dashed points). Data highlighted for the cluster G1 (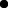), G2 (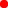), or G3 (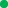) they belong to and their respective barycenter (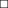, 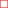, 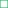).

The general features observed for the patterns of lignocellulosic enzyme activities among BRFs and WRFs emphasize differences in the mechanisms involved in the lignocellulose breakdown between these two fungal groups. The hierarchical clustering based on ligno- and holocellulolytic activities classified the strains into two major groups for both substrates (Fig. 4). The larger group contains mainly WRFs belonging to the three clusters G1, G2 and G3 (except for *Antrodia malicola* BRFM 1200, *Postia stiptica* BRFM 1148, *Fomitopsis pinicola* BRFM 1291 and *Fomitopsis rosea* BRFM 1062), while the second group contains only BRFs belonging to G2.

Despite the general features observed in the patterns of extracellular enzyme activities among BRFs and WRFs, some strains produce distinct enzymatic profiles. The BRFs *A. malicola* BRFM 1200 and *P. stiptica* BRFM 1148 show an enzymatic pattern close to the WRFs presenting low carbohydrate hydrolysing and ligninolytic activities as described elsewhere (Huang *et al*., 2009; Wei *et al*., 2010). The two BRF strains *F. pinicola* BRFM 1291 and *F. rosea* BRFM 1062 produce carbohydrate hydrolysing enzymes with lower levels compared with most of the BRFs.

Regardless of the cluster the strains belong to, the fungal enzymatic profiles were not correlated with saccharification efficiency. However, it must be kept in mind that lignocellulose breakdown occurs through complex mechanisms involving an arsenal of plant cell wall-degrading enzymes. The lignocellulolytic activities studied here give only a snapshot of this arsenal (only one time point at day 12); more detailed investigations to take into account the greater complexity of the multi-enzymatic process are necessary to increase our knowledge and understanding of the relationship between the fungal lignocellulolytic enzyme system and lignocellulose breakdown.

This study highlights the importance of a systematic assessment of fungal biodiversity in order to identify promising fungal strains for the conversion of lignocellulosic biomass. The new and efficient multi-well plate SSF method developed here copes with the necessity of screening hundreds of fungi on different biomass feedstocks. To our knowledge, this is the first report of SSF culturing of filamentous fungi in a multi-well format with *in situ* post-treatments at a scale suitable for medium-throughput screening. The ability to assess rapidly, accurately and reproducibly a wider range of fungi represents a breakthrough to better explore and exploit fungal biodiversity in a biorefinery concept to produce biofuels and value-added bioproducts.

## Conclusions

A large number of WRFs and BRFs were simultaneously evaluated for lignocellulosic biomass conversion by a multi-well plate SSF method. Several fungi, mainly WRFs, were found to show high potential for biological pretreatments, as they enhanced enzymatic hydrolysis. A few strains were found to be efficient on both wheat straw and miscanthus, which makes them promising candidates deserving further investigation. The medium-throughput screening method described in this study is a powerful tool to preselect a restricted number of strains suited for a natural raw material/relevant industrial application combination. The preselected strains can then undergo scaled-up SSF to further select the best-adapted ones and to optimize the process. For this purpose, critical factors, not provided at a small scale, such as weight loss, carbohydrate preservation, quantification of fungal biomass, etc., need to be taken into account.

The scaled down SSF method developed in this study could be useful to preselect relevant fungi for biorefining in White and Green Chemistry, and could be applied to a wide variety of microorganisms and substrates. The method could also be helpful for performing preliminary assays for culture condition optimization.

## Experimental procedures

### Fungal strains

The basidiomycetes fungi used in the present study (Table S1) were obtained from the CIRM-CF dedicated to filamentous fungi of biotechnological interest (http://www.inra.fr/crb-cirm/) at the French National Institute for Agricultural Research (Marseille, France). All the strains were authenticated by ITS sequencing. The screened fungi were isolated from tropical and temperate forests. As proposed by Welti and colleagues (2012), three tropical species of ‘*Trametes*’: *T. menziesii* BRFM 1369 & BRFM 1557, *Trametes lactinea* BRFM 1251 & BRFM 1282 and *Leiotrametes sp.* BRFM 1048 & BRFM 1053 (Banque de Ressources Fongiques de Marseille) were classified in the genus *Leiotrametes*. Two specific strains *Ganoderma weberianum* BRFM 1167 and *Trametes meyenii* BRFM 1170 were initially obtained from the Centraalbureau voor Schimmelcultures (Baarn, The Netherlands) and the American Type Culture Collection (Manassas, Virginia, USA) respectively. The fungal species were maintained on 2% malt extract agar slants at 4 °C.

### Raw material

Wheat straw (Haussmann soft wheat) and *Miscanthus giganteus* stem were obtained from VIVESCIA (Reims, France), naturally dried and chopped (≈ 4 mm, Cutting Mill SM 100, Retsch®, Germany).

### Growth conditions

Miniaturized fungal pretreatment cultures were performed in polypropylene 24-well plates (Whatman INC, Piscataway, USA) under SSF conditions. Wheat straw (0.1 g, dry weight) or miscanthus and 4 ml deionized water were dispensed into each well of the plates. The plates were sealed with aluminum foil by an automatic plate sealer (PlateLoc, Agilent, USA) and autoclaved at 110 °C for 30 min. After sterilization, the plates were centrifuged at 3500 r.p.m. for 5 min, and 2 ml of the supernatant were discarded. The substrate was then washed by adding 2 ml of sterilized water to each well, stirring at 450 r.p.m. for 10 min (Microtron, Infors AG, Switzerland) and removing 2 ml of the wash liquid after subsequent centrifuging. After repeating the washing steps three times, 2 ml of nutrient solution (40 g l^−1^ glucose and 3.68 g l^−1^ diammonium tartrate) were added per well and mixed. The excess liquid medium (i.e. 3 ml) was removed, leading to a final lignocellulosic substrate consistency of 10% dm (w/v). Three non-inoculated controls per plate were set up by replacing the nutrient solution with 2 ml of water. Finally, wells were inoculated in triplicate for each fungal strain, with one 5 mm diameter agar disc of 7-day-old mycelia crushed and mixed with substrates using a sterilized toothpick. The plates were covered with an adhesive membrane (Breathseal, Greiner Bio-One), which allows gas exchange, and were incubated at 25 °C for 12 days under saturated humidity conditions. To allow an extensive fungal colonization on the substrates, the substrates embedded in fungal mycelium were turned upside down at day 6, leaving an air space at the bottom of the well.

### Fungal post-treatments

All fungal post-treatments described below were performed *in situ* in the same 24-well plate culture (Fig. 1).

#### Extraction of water-soluble components

Biopretreated and non-inoculated substrates after 12 days of incubation were washed with deionized water (1.5 ml well^−1^) for 2 h, at 50 °C and 450 r.p.m. (Microtron) and then centrifuged at 2500 r.p.m. for 5 min. Three hundred microlitre aliquots of supernatants were harvested and dispensed into a 96-well filter-plate (1 μm) to be centrifuge-filtered (2500 r.p.m.; 5 min) for further analyses.

#### Mild alkaline wash

The remaining solid fractions underwent mild alkali treatments as described by Salvachua and colleagues (2011), adapted to a 24-well plate with 0.1% (w/v) sodium hydroxide with a substrate consistency of 5% dm (w/v) at 50 °C and 450 r.p.m. for 1 h. The substrate was then washed by adding 3 ml of deionized water to each well, incubating at 50 °C and 450 r.p.m. for 10 min (Microtron) and removing 3 ml of the wash liquid after centrifugation at 3500 r.p.m. for 5 min. The washing steps were repeated four times to reach neutral pH in the last water wash.

#### Enzymatic hydrolysis assays

Experiments were carried out at 2.5% dm (w/v) consistency by adding 3 ml of citrate phosphate buffer pH 4.8, 50 mM. The suspension was further supplemented with 12 FPU g^−1^ substrate (dry weight) of commercial cellulases GC220 from *T. reesei* (Genencor Danisco, NY, USA) and 60 U g^−1^ substrate (dry weight) of β-glucosidase from *A. niger* (Novozyme SP188). Tetracycline (150 mg l^−1^) and cycloheximide (40 mg l^−1^) were added to prevent any microbial contamination. The 24-well plates were then heat sealed with aluminum using a plate sealer (PlateLoc), and incubated at 50 °C and 450 r.p.m. in a Microtron incubator for 72 h. The 24-well plates were centrifuged at 3500 r.p.m. for 10 min, and the resulting hydrolysis supernatants (250 μl) were taken from the reaction mixture and dispensed into 96-well plates. The reducing sugars and the glucose released were quantified respectively using the dinitrosalicylic acid method (Navarro *et al*., 2010) and the Glucose RTU kit (Biomérieux, Marcy-l'Etoile, France). All assays were carried out in triplicate. For all experiments, the variation coefficient was lower than 10%.

Glucose and reducing sugar yields are expressed as quantity of glucose or reducing sugars released per gram of initial dry substrate. To calculate the percentage improvement, glucose and reducing sugar yields of non-inoculated controls were considered as 100%.

### Enzyme activity assays

Endo-1,4-β-glucanase and endo-1,4-β-xylanase activities were assessed in a 96-well plate by measuring, respectively, the hydrolysis of 1% azo-CM-cellulose and 1% azo-xylan (Megazyme, Ireland) in citrate phosphate buffer pH 4.8, 50 mM. Ten microlitres of the water extract were added to 50 μl of azo-substrate solutions. The 96-well plates were then heat sealed with aluminum (PlateLoc) and incubated at 50 °C for 3 h. One hundred fifty microlitres of precipitant solution at pH 5 [80% (v/v) ethanol with 0.29 M sodium acetate and 18 mM zinc acetate] were then added to precipitate undigested cellulose or xylan. The plates were stirred, incubated for 10 min at room temperature and centrifuged at 3500 r.p.m. for 5 min. Supernatants were collected and the optical density was measured at 590 nm. Activities were expressed as percentage of azo-substrate hydrolysed with the 100% value taken as the mean absorbance at 590 nm of three wells to which no ethanol and no water extract had been added.

The laccase and peroxidase activities were determined using 2,2′-azino-bis(3-ethylbenzthiazoline-6-sulphonic acid) (ABTS) and 2,6-dimethoxyphenol (DMP) as substrates respectively. All tests were conducted in 96-well plates. For laccase activity, the reaction mixture (total volume 210 μl) contained 20 μl of water extract and 0.476 mM of ABTS in 47.6 mM sodium tartrate buffer at pH 4.0. The reaction was monitored by measuring ABTS oxidation at 420 nm and 30 °C for 0.5 min. For peroxidase activity, the reaction mixture (total volume 200 μl) contained 10 μl of water extract, 5 mM DMP in 100 mM sodium tartrate buffer, pH 5.0, 0.10 mM MnSO_4_ and 4 mM of sodium fluoride (laccase inhibitor) with or without 0.1 mM H_2_O_2_. The increase in absorption at 469 nm was monitored at 30 °C for 5 min. The activities of peroxidases were obtained by subtracting values obtained in the presence and absence of H_2_O_2_. Ligninolytic enzyme activities were expressed in nkatal ml^−1^, where one nkatal is defined as the amount of enzyme that oxidizes 1 nmole of substrate per second.

### Statistical analysis

PCA and HCA were performed using the FactoMineR package in the R commander environment with R (R Development Core Team 2007), available from the Comprehensive R Archive Network at http://CRAN.R-project.org/.

## Supporting Information

**Table S1.** Fungal strains from the CIRM collection screened in this study.
