# Supplementary material for: Solid-state fermentation in multi-well plates to assess pretreatment efficiency of rot fungi on lignocellulose biomass
Source: Microb Biotechnol. 2015 Aug 6;8(6):940–9. doi: 10.1111/1751-7915.12307 (PMC4621447; doi:10.1111/1751-7915.12307)
Supplement: Supplementary file 1 — Table S1. Fungal strains from the CIRM collection screened in this study. [file mbt20008-0940-sd1.docx]

**Table 1:** Fungal strains from the CIRM collection screened in this study.

| **Strains** | **Geography (Continent > Country > Province)** | **Rot type** | **Family** | **BRFM**  **Number** |
| --- | --- | --- | --- | --- |
|  |  |  |  |  |
|  | **Tropical forests** |  |  |  |
|  |  |  |  |  |
| *Artolenzites elegans* | South America > French Guiana > Saül | WRF | *Polyporaceae* | 1122 |
| *Artolenzites elegans* | Oceania > New Caledonia > NA | WRF | *Polyporaceae* | 1280 |
| *Ganoderma flaviporum* | South America > Martinique (Island) > NA | WRF | *Ganodermataceae* | 1570 |
| *Ganoderma sp.* | South America > French Guiana > Saül | WRF | *Ganodermataceae* | 1033 |
| *Ganoderma sp.* | South America > French Guiana > Saül | WRF | *Ganodermataceae* | 1036 |
| *Ganoderma sp.* | South America > French Guiana > Sinnamary | WRF | *Ganodermataceae* | 1030 |
| *Ganoderma subfornicatum* | South America > French Guiana > Kourou | WRF | *Ganodermataceae* | 1029 |
| *Ganoderma subfornicatum* | South America > French Guiana > Regina | WRF | *Ganodermataceae* | 1248 |
| *Ganoderma subfornicatum* | South America > French Guiana > Saül | WRF | *Ganodermataceae* | 1026 |
| *Ganoderma subfornicatum* | South America > French Guiana > Sinnamary | WRF | *Ganodermataceae* | 1241 |
| *Ganoderma weberianum* | Asia > Philippines > NA | WRF | *Ganodermataceae* | 1167 |
| *Grammothele sp.* | South America > French Guiana > NA | WRF | *Polyporaceae* | 910 |
| *Grammothele sp.* | South America > French Guiana > NA | WRF | *Polyporaceae* | 931 |
| *Humphreya coffeata* | South America > Martinique (Island) > NA | WRF | *Ganodermataceae* | 1541 |
| *Leiotrametes sp.* | South America > French Guiana > Kourou | WRF | *Polyporaceae* | 1048 |
| *Leiotrametes sp.* | South America > French Guiana > Saül | WRF | *Polyporaceae* | 1053 |
| *Lenzites sp.* | South America > French Guiana > NA | WRF | *Polyporaceae* | 1347 |
| *Lenzites sp.* | South America > French Guiana > Saül | WRF | *Polyporaceae* | 1052 |
| *Phlebia sp.* | South America > French Guiana > Macouria | WRF | *Meruliaceae* | 1123 |
| *Pycnoporus sanguineus* | Oceania > New Caledonia > NA | WRF | *Polyporaceae* | 980 |
| *Trametes cingulata* | Africa > Malawi > NA | WRF | *Polyporaceae* | 1296 |
| *(Leio)Trametes lactinea* | South America > French Guiana > Regina | WRF | *Polyporaceae* | 1251 |
| *(Leio) Trametes lactinea* | Oceania > New Caledonia > NA | WRF | *Polyporaceae* | 1282 |
| *Trametes maxima* | South America > Martinique (Island) > NA | WRF | *Polyporaceae* | 1555 |
| *(Leio) Trametes menziesii* | South America > Martinique (Island) > NA | WRF | *Polyporaceae* | 1369 |
| *(Leio) Trametes menziesii* | South America > Martinique (Island) > NA | WRF | *Polyporaceae* | 1557 |
| *Trametes meyenii* | Asia > India > NA | WRF | *Polyporaceae* | 1170 |
| *Trametes mimetes* | Africa > Zimbabwe > NA | WRF | *Polyporaceae* | 1295 |
| *Trametes pavonia* | South America > Martinique (Island) > NA | WRF | *Polyporaceae* | 1554 |
| *Trametes polyzona* | Africa > Zimbabwe > NA | WRF | *Polyporaceae* | 1183 |
| *Trametes sp.* | South America > French Guiana > NA | WRF | *Polyporaceae* | 1361 |
|  |  |  |  |  |
|  | **Temperate forests** |  |  |  |
|  |  |  |  |  |
| *Abortiporus biennis* | Europe > France > Orne | WRF | *Meruliaceae* | 1215 |
| *Antrodia malicola* | Europe > France > Rhone-Alpes | BRF | *Fomitopsidaceae* | 1200 |
| *Daedalea quercina* | Europe > France > Orne | BRF | *Fomitopsidaceae* | 877 |
| *Dichostereum effuscatum* | Europe > France > NA | WRF | *Lachnocladiaceae* | 91 |
| *Fomes fomentarius* | Europe > France > Corsica | WRF | *Polyporaceae* | 1323 |
| *Fomitopsis iberica* | Europe > France > Corsica | BRF | *Fomitopsidaceae* | 1308 |
| *Fomitopsis pinicola* | Europe > France > Vaucluse | BRF | *Fomitopsidaceae* | 1197 |
| *Fomitopsis pinicola* | Europe > Lithuania > NA | BRF | *Fomitopsidaceae* | 1291 |
| *Fomitopsis rosea* | Europe > France > NA | BRF | *Fomitopsidaceae* | 1062 |
| *Fomitopsis spraguei* | Europe > France > NA | BRF | *Fomitopsidaceae* | 665 |
| *Ganoderma adspersum* | Europe > France > Var | WRF | *Ganodermataceae* | 1161 |
| *Ganoderma applanatum* | Europe > France > Isère | WRF | *Ganodermataceae* | 883 |
| *Ganoderma lucidum* | Europe > France > Bouches du Rhone | WRF | *Ganodermataceae* | 885 |
| *Gloeophyllum trabeum* | Europe > Germany > NA | BRF | *Gloeophyllaceae* | 236 |
| *Grifola frondosa* | Europe > France > Orne | WRF | *Meripilaceae* | 1162 |
| *Hexagonia nitida* | Europe > France > Vaucluse | WRF | *Polyporaceae* | 1328 |
| *Inonotus radiatus* | Europe > France > Orne | WRF | *Hymenochaetaceae* | 1193 |
| *Ischnoderma benzoinum* | Europe > France > Aude | WRF | *Fomitopsidaceae* | 1133 |
| *Lenzites warnieri* | Europe > France > NA | WRF | *Polyporaceae* | 973 |
| *Piptoporus betulinus* | Europe > France > NA | BRF | *Fomitopsidaceae* | 967 |
| *Pleurotus ostreatus* | Europe > France > Corsica | WRF | *Pleurotaceae* | 1326 |
| *Polyporus brumalis* | Europe > France > NA | WRF | *Polyporaceae* | 985 |
| *Postia stiptica* | Europe > France > Var | BRF | *Fomitopsidaceae* | 1148 |
| *Pycnoporus cinnabarinus* | Europe > France > NA | WRF | *Polyporaceae* | 1297 |
| *Pycnoporus coccineus* | Asia > Japan > NA | WRF | *Polyporaceae* | 46 |
| *Pycnoporus coccineus* | Oceania > Australia > NA | WRF | *Polyporaceae* | 1396 |
| *Stereum hirsutum* | Europe > France > Bouches du Rhone | WRF | *Stereaceae* | 889 |
| *Trametella trogii* | Europe > France > NA | WRF | *Polyporaceae* | 974 |
| *Trametes gibbosa* | Europe > France > NA | WRF | *Polyporaceae* | 952 |
| *Trametes gibbosa* | Europe > France > Orne | WRF | *Polyporaceae* | 1115 |
| *Trametes ljubarskii* | Europe > France > NA | WRF | *Polyporaceae* | 957 |
| *Trametes suaveolens* | Europe > France > NA | WRF | *Polyporaceae* | 578 |
|  |  |  |  |  |

NA, not available.
